# PriPath: identifying dysregulated pathways from differential gene expression via grouping, scoring, and modeling with an embedded feature selection approach

**DOI:** 10.1186/s12859-023-05187-2

**Published:** 2023-02-23

**Authors:** Malik Yousef, Fatma Ozdemir, Amhar Jaber, Jens Allmer, Burcu Bakir-Gungor

**Affiliations:** 1 Department of Information Systems, Zefat Academic College, 13206 Zefat, Israel; 2 Galilee Digital Health Research Center (GDH), Zefat Academic College, Zefat, Israel; 3 Department of Computer Engineering, Faculty of Engineering, Abdullah Gul University, Kayseri, Turkey; 4 University Institute of Digital Communication Systems, Ruhr-University, Bochum, Germany; 5 Medical Informatics and Bioinformatics, Institute for Measurement Engineering and Sensor Technology, Hochschule Ruhr West, University of Applied Sciences, Mülheim an der Ruhr, Germany

**Keywords:** Feature selection, Feature scoring, Feature grouping, Biological knowledge integration, KEGG pathway, Classification, Gene expression, Enrichment analysis, Machine learning, Bioinformatics, Data science, Data mining, Genomics

## Abstract

**Background:**

Cell homeostasis relies on the concerted actions of genes, and dysregulated genes can lead to diseases. In living organisms, genes or their products do not act alone but within networks. Subsets of these networks can be viewed as modules that provide specific functionality to an organism. The Kyoto encyclopedia of genes and genomes (KEGG) systematically analyzes gene functions, proteins, and molecules and combines them into pathways. Measurements of gene expression (e.g., RNA-seq data) can be mapped to KEGG pathways to determine which modules are affected or dysregulated in the disease. However, genes acting in multiple pathways and other inherent issues complicate such analyses. Many current approaches may only employ gene expression data and need to pay more attention to some of the existing knowledge stored in KEGG pathways for detecting dysregulated pathways. New methods that consider more precompiled information are required for a more holistic association between gene expression and diseases.

**Results:**

PriPath is a novel approach that transfers the generic process of grouping and scoring, followed by modeling to analyze gene expression with KEGG pathways. In PriPath, KEGG pathways are utilized as the grouping function as part of a machine learning algorithm for selecting the most significant KEGG pathways. A machine learning model is trained to differentiate between diseases and controls using those groups. We have tested PriPath on 13 gene expression datasets of various cancers and other diseases. Our proposed approach successfully assigned biologically and clinically relevant KEGG terms to the samples based on the differentially expressed genes. We have comparatively evaluated the performance of PriPath against other tools, which are similar in their merit. For each dataset, we manually confirmed the top results of PriPath in the literature and found that most predictions can be supported by previous experimental research.

**Conclusions:**

PriPath can thus aid in determining dysregulated pathways, which applies to medical diagnostics. In the future, we aim to advance this approach so that it can perform patient stratification based on gene expression and identify druggable targets. Thereby, we cover two aspects of precision medicine.

**Supplementary Information:**

The online version contains supplementary material available at 10.1186/s12859-023-05187-2.

## Background

Today, the healthcare system is facing a shift toward precision medicine. When diseases are evaluated at the molecular level, patient stratification becomes possible so that the most suitable medication can be identified more individually. This approach heavily depends on the molecular data obtained through recent high-throughput methods. For example, next-generation sequencing technologies are utilized to analyze genome diversity, epigenomics, and gene-expression profiling [1, 2]. Along this line, transcriptomic data are generated at an unprecedented pace. Much of these data adhere to the FAIR (Findable, Accessible, Interoperable, and Reusable) principles; hence, large gene expression data sets have become publicly available concerning various diseases.

The current bottleneck is in biomedical data analysis, starting from the preprocessing of the sequencing data to supporting decision-making processes, e.g., drug selection. The high dimensionality of the data (a large number of genes) combined with a small number of samples makes it difficult to interpret the data. In this respect, feature selection is essential for dimensionality reduction and selecting the most informative genes. Additionally, more efficient gene selection methods are proposed to achieve the full potential of the growing data pool, develop gene-based diagnostic tests, and aid drug discovery. Integrative gene selection incorporates domain knowledge from external biological resources such as databases to improve the gene selection approaches [3]. Gene ontology (GO) is one resource used to integrate biological background information with the analysis of gene expression data.

Genes do not act alone to perform their biological functions but are organized into, for example, metabolic and signaling pathways. These can be regarded as groups of genes. The pathway information can be exploited for feature selection in gene expression data analysis. In other words, the grouping of the genes in terms of pathways can be incorporated into the feature selection problem to identify gene expression signatures. It has been shown that for gene expression data analysis, the methods incorporating pathway knowledge usually outperform their gene-based counterparts where biological domain knowledge or pathway knowledge is not considered [4]. While the traditional methods rely on identifying statistically significant differentially expressed genes between two different phenotypes, pathway knowledge-based methods impose further constraints on the prediction task and force training methods to choose more meaningful genes. KEGG is a frequently used external pathway database [5], and it has recently been enriched with new pathways, cellular processes, and diseases [6].

The integration of pathway knowledge can vary among different methods. While some algorithms treat pathways as a graph, consider the underlying topology of the pathways, and analyze the connections of genes, other methods consider the pathways as gene sets. A comprehensive review of topology-based (TB) vs. non-topology-based (non-TB) pathway analysis methods can be found in [6]. Comparative evaluation of topology-based pathway enrichment analysis methods can also be found in [7, 8]. Non-topology-based pathway-guided gene selection methods treat every gene inside a specific pathway equally and assign equal weights.

On the other hand, topology-based pathway-guided gene selection methods compute the connectivity level of the genes inside a pathway and use this information while weighting the genes. Hence, in topology-based and pathway-guided gene selection methods, genes having high connectivity in a pathway may be prioritized. There are also functional score-based gene selection methods, such as [3, 9], which consider only the pathway membership of the genes to generate an evaluation score. These methods implicitly assume that all genes belonging to a specific pathway co-regulate and co-function. Other approaches to gene set enrichment analysis (GSEA) employ different grouping factors for genes, such as function or chromosomal proximity [10].

Regarding predictive accuracy, some studies, such as [11, 12], have concluded that pathway-guided gene selection methods do not outperform classic gene-based feature selection methods. This performance difference may be explained by the fact that the pathway knowledge retrieved from pathway databases such as KEGG [13], Gene Ontology [14], and Reactome [15] conveys no or limited meaningful information for some datasets. In contrast, the pathways constructed in a “data-driven” way may be more informative for the diseases under investigation and thus preferred over the canonical pathways.

A recent review [16] presented machine learning-based approaches for integrating biological domain knowledge into gene expression data analysis. The authors discussed several tools that adopt the so-called Grouping-Scoring-Modeling (G–S–M) approach. For instance, maTE [17] integrates biological knowledge of microRNAs (miRNA) for grouping genes. CogNet [18] performs KEGG pathway enrichment analysis based on ranked active subnetworks. MiRcorrNet [19] detects groups of miRNA-mRNAs by analyzing the correlation between miRNA and mRNA expression profiles obtained from the same sample.

Similarly, miRModuleNet [20] detects miRNA-mRNA regulatory modules to serve as groups while analyzing two omics datasets. Another G–S–M model-based study by Yousef et al. [21] utilizes GO terms for grouping the genes. GediNET [22] also discovers disease-gene associations using the G–S–M model. The first study, which considered grouped gene clusters rather than individual genes, was also developed by Yousef et al. [23, 24]. The tools mentioned above are different implementations of this idea for various data types. SVM-RCE (Support Vector Machines Recursive Cluster Elimination) groups genes based on their gene expression values [23]. It scores each cluster of genes by incorporating a machine-learning algorithm. This approach has received attention from other researchers.

Similarly, SVM-RNE [25] is based on gene network detection to serve as groups for scoring by the G–S–M model. SVM-RCE-R is one other example developed along this line. However, there is still room for developing more tools based on the G–S–M model and incorporating biological knowledge, such as KEGG pathways.

In this paper, we introduce a novel tool named PriPath, which ranks and groups biological information based on the G–S–M model. PriPath treats KEGG pathways as gene sets, neglecting the structure of the pathway. Instead, the algorithm uses KEGG pathways for grouping the genes to perform scoring and classification. PriPath produces performance metrics and a list of dysregulated KEGG pathways for the condition under study. The innovation of our approach stems from its ability to search the space of the groups of the KEGG pathway to rank and find the most important groups.

We have tested PriPath on 13 gene expression datasets of various cancers and other diseases. The results indicate that PriPath outperforms maTE in most cases in terms of classifier performance and uses less number of genes than SVM-RCE-R and CogNet. Additionally, for each dataset, we compared PriPath predictions to the predictions of Reactome and DAVID and manually assessed the top 3 PriPath predictions in the literature. PriPath could detect biologically and clinically relevant pathways. PriPath can assist in identifying dysregulated pathways, which is applicable in medical diagnostics. Hence, we tackle an aspect of precision medicine.

The rest of the manuscript is organized as follows. “Materials” and “Methods” Sections describe the materials and methods used. “Results” Section evaluates the results by comparing the proposed approach with other embedded feature selection and enrichment tools using different approaches. “Discussions” Section discusses the top predictions considering literature evidence, and “Conclusions” Section concludes the work.

## Materials

### Gene expression data

To support algorithm development and testing, 13 human gene expression datasets (as shown in detail in Table 1) were downloaded from the Gene Expression Omnibus (GEO) [26] at NCBI. Disease (positive) and control (negative) data were acquired for all datasets. 13 datasets were used to test PriPath and were used for qualitative comparison with other tools. Moreover, these 13 datasets enabled performance comparison with tools that previously utilized the same data, such as maTE and CogNet.

**Table 1:** Characteristics of the 13 gene expression datasets used in this study

GEO accession	Title	Disease	Number of samples	Number of samples in classes
GDS1962	Glioma-derived stem cell factor effect on angiogenesis in the brain	Glioma	180	Negative = 23 positive = 157
GDS2547	Metastatic prostate cancer (HG-U95C)	Prostate cancer	164	Negative = 75 positive = 89
GDS4824	Prostate cancer Analysis of malignant and benign prostate tissues	Prostate cancer	21	Negative = 8 positive = 13
GDS3268	Colon epithelial biopsies of ulcerative colitis patients	Colitis	202	Negative = 73 positive = 129
GDS3646	Celiac disease: primary leukocytes	Celiac disease	132	Negative = 22 positive = 110
GDS3874	Diabetic children: peripheral blood mononuclear cells (U133A)	Diabetes	117	Negative = 24 positive = 93
GDS3875	Diabetic children: peripheral blood mononuclear cells (U133B)	Diabetes	117	Negative = 24 positive = 93
GDS5037	Severe asthma: bronchial epithelial cell	Asthma	108	Negative = 20 positive = 88
GDS5499	Pulmonary hypertension: PBMCs	Pulmonary hypertension	140	Negative = 41 positive = 99
GDS3837	Non-small cell lung carcinoma in female nonsmokers	Lung cancer	120	Negative = 60 positive = 60
GDS4516 GDS4718	Colorectal cancer: laser microdissected tumor tissues (homogenized)	Colorectal cancer	148	Negative = 44 positive = 104
GDS2609	Early onset colorectal cancer: normal-appearing colonic mucosa	Colorectal cancer	22	Negative = 10 positive = 12
GDS3794	Rheumatoid arthritis: peripheral blood mononuclear cells	Arthritis	33	Negative = 15 positive = 18

### KEGG data

We downloaded the KEGG data from Bioconductor using the R programming language [27] on 21.01.2021. The KEGG data contains 32,083 entries representing 331 KEGG pathways [28].

## Methods

### Algorithm

**Fig. 1:**
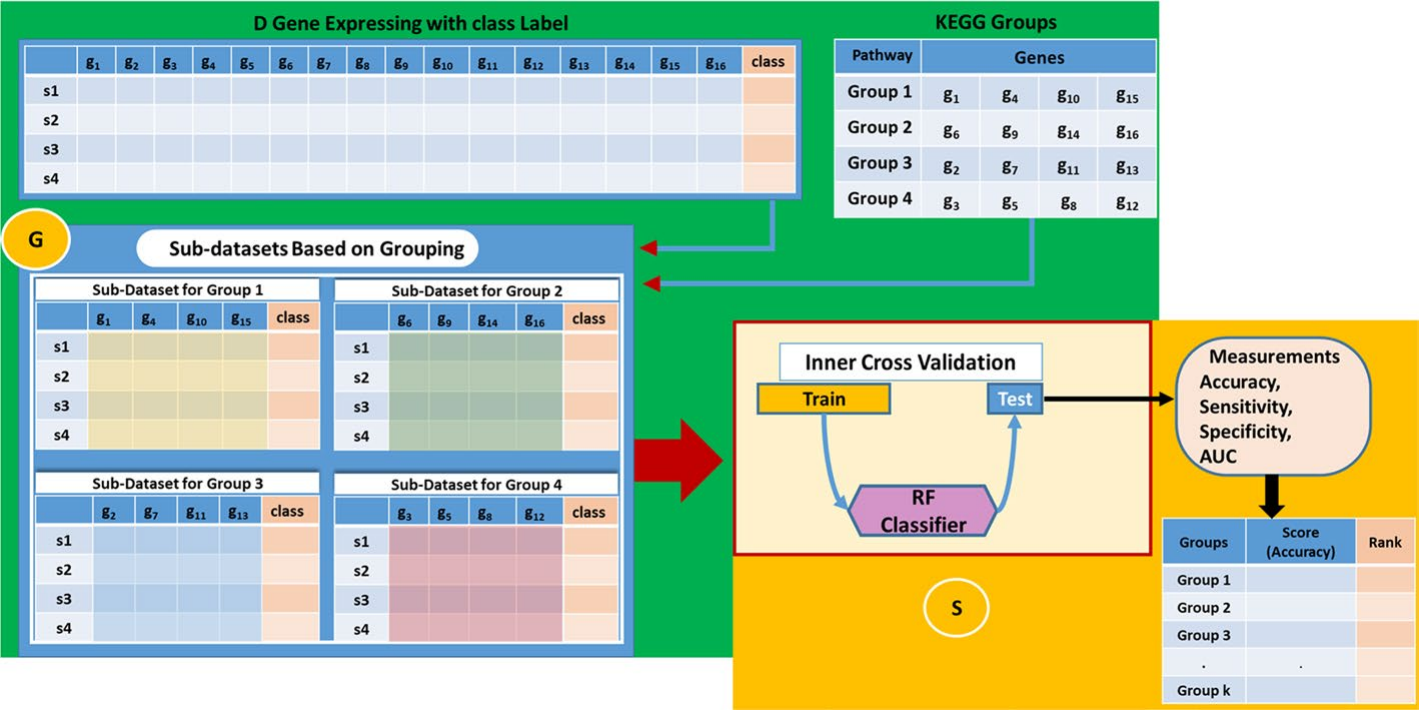
The flowchart of the PriPath algorithm. The green panel corresponds to the Grouping (G) component. The gold panel corresponds to the Scoring (S) component. The G component uses the D matrix and the KEGG groups to create sub-datasets for each group. The S component performs inner cross-validation for each sub-dataset and reports performance metrics

PriPath employs machine learning to determine which KEGG pathways are associated with differences in gene expression between control and disease samples. PriPath aims to find dysregulated KEGG pathways for the disease under study using gene expression data and the Grouping, Scoring, and Modeling (G–S–M) approach. Following the G–S–M approach proposed in [16], the PriPath algorithm contains three main components. Figure 1 presents the general flowchart of the proposed algorithm.

Let D (e.g., the training data shown in Fig. 1) be the gene expression data represented as a matrix, where *s* denotes the samples. For simplicity, we consider that the genes are the columns of the matrix, while the samples are the rows. The matrix D contains a special column called *label,* which indicates the class annotation for each row. The class labels could be disease or any experimental condition versus a control. The test data (shown in Fig. 1) is kept out of the G–S–M approach and only used to test the final model.

The PriPath algorithm requires two inputs, KEGG pathways, and gene expression data. We consider KEGG pathways as groups of genes such that each KEGG pathway is represented by a set of genes (i.e., multiple occurrences of one gene in a pathway are ignored). Table 2 is an example of the KEGG pathways groups. Let us assume that we have *k* such groups. Let *kgg_grp* = {*g*_5_, *g*_12_, *g*_18_, *g*_22_} represent one KEGG group that consists of *5* genes.

**Table 2:** An example of KEGG groups with their associated genes

KEGG pathway ID	Group name	Genes	References
hsa05220	Chronic myeloid leukemia	TP53, MYC, HRAS, KRAS, …	[29]
hsa00670	One carbon pool by folate	MTHFD2, DHFR, DHFR2, MTHFD1L, …	[30]
hsa05033	Nicotine addiction	GABRA5, CACNA1B, GABRE, GABRD, …	[31]
hsa05200	Cancer	GNG4, CTNNA1, TP53, MYC, …	[32]
hsa05216	Thyroid cancer	TP53, MYC, TCF7L1, MAPK1, …	[33]
hsa04723	Retrograde endocannabinoid signaling	GNG4, MAPK9, PRKCG, GABRA5,…	[34]

The first column is the KEGG pathway ID, the second column refers to the KEGG pathway name (group name), and the third column is the set of genes associated with the KEGG pathway

The differential expressions of each gene between control and disease are detected by a t-test applied on D, the training dataset, during the preprocessing step of the tool. The t-test statistics are considered for the next steps of the algorithm. The algorithm of how PriPath utilizes these input data for its predictions is illustrated in Fig. 1. An essential step of the PriPath tool is the Grouping component, G (Fig. 1, green panel). The G component utilizes the KEGG groups to create k-related sub-datasets, where each sub-dataset is extracted from the primary data D. The green panel in Fig. 1 depicts the process of creating sub-datasets. Those k sub-datasets are then subjected to the Scoring Component, S (Fig. 1, gold panel).

A sub-dataset is created from the output of the G component (*kgg_grp* = {*g*_5_, *g*_12_, *g*_18_, *g*_22_}) and the associated input D. Its columns will be the corresponding columns of g5, g12, g18, and g22; and its rows are all the sample rows with the associated class labels. This creates a sub-dataset that can be subjected to any machine learning algorithm. The green panel in Fig. 1 presents an example with the D matrix and four KEGG groups to create four sub-datasets, each corresponding to one group. Those four sub-datasets will serve as input to the S component.

The Scoring component S (Fig. 1, gold panel) iterates over all sub-datasets to assign a score representing the capacity to separate the two classes considering just the genes associated with the group. Since we observed that the Random Forest (RF) classifier performed well in our previous studies [17, 18], we used RF in this study to perform the scoring. But the overall data analysis workflow is so flexible that any other classifier could replace RF. Replacing the RF classifier in the implementation of PriPath can be done by the users via replacing the RF node with, for example, the support vector machine node in KNIME. The inner cross-validation is applied by splitting the sub-dataset into two parts, one for training the RF and the other for testing. We repeat this procedure r times (here: r = 5). The accuracy, sensitivity, specificity, and other statistics are collected and reported as their average over the cross-validation. The accuracy average serves as the score of each group/sub-dataset.

In the Modeling component M (not shown in Fig. 1), for each KEGG pathway as the grouping factor, the best j KEGG pathways are selected, and their genes are combined. An RF model is trained with the grouping function given by the top-ranked j pathways. Finally, the model is tested on the test data, and the performance statistics are reported (in our case: $j\in \left\{1..10\right\}$). Training and testing were performed using 80% and 20% of the data. However, such settings can be adjusted in our KNIME implementation of PriPath.

#### Classification approach

We utilized the random forest classifier implemented by the KNIME platform [35]. For each model creation, 80% of the data (training data) was used to train the classifier, and 20% of the data (testing data) was used to test the classifier. The data sets are imbalanced in terms of the two classes. This imbalance can affect the performance of the trained classifier. To address this issue, we employed random under-sampling of the majority class. During model creation, under-sampling is incorporated into the tenfold Monte Carlo cross-validation (MCCV) [36] approach. The default parameters were used for RF training. The number of levels (tree depth) was not limited, and the number of models was set to 100.

#### Model performance evaluation

To compare among trained models, several statistical measures such as sensitivity, specificity, and accuracy were calculated [37]. The following formulations were calculated from the confusion matrix (TP, true positive; FP, false positive; TN, true negative; and FN, false negative).

$${\text{Sensitivity}}\,\left( {{\text{SE}},\,\,{\text{Recall}}} \right)\,\, = \,\,{\text{TP}}/\left( {{\text{TP}}\, + \,{\text{FN}}} \right)$$

$${\text{Specificity}}\,\left( {{\text{SP}}} \right)\, = \,{\text{TN}}/\left( {{\text{TN}}\, + \,{\text{FP}}} \right)$$

$${\text{Accuracy}}\,\left( {{\text{ACC}}} \right)\, = \,\left( {{\text{TP}}\, + \,{\text{TN}}} \right)/\left( {{\text{TP}}\, + \,{\text{TN}} + \,{\text{FP}}\, + \,{\text{FN}}} \right)\,$$

In addition, the area under the receiver operator characteristic (ROC) curve (AUC), which assesses the probability that a classifier will rank a randomly selected positive sample higher than a randomly selected negative sample [38], was calculated. All performance measures are presented as the average of tenfold MCCV if not otherwise stated.

### Implementation

We utilized the Konstanz information miner (KNIME) [35] to implement the PriPath algorithm. For the implementation part of our algorithm, we have decided to utilize the free and open-source platform KNIME since it is simple to use and provides user-friendly graphical representations. KNIME has also been employed to develop numerous bioinformatics data analysis workflows. KNIME workflows include processing nodes and data links (edges). In the workflow, edges provide data transport from one node to another.

**Fig. 2:**
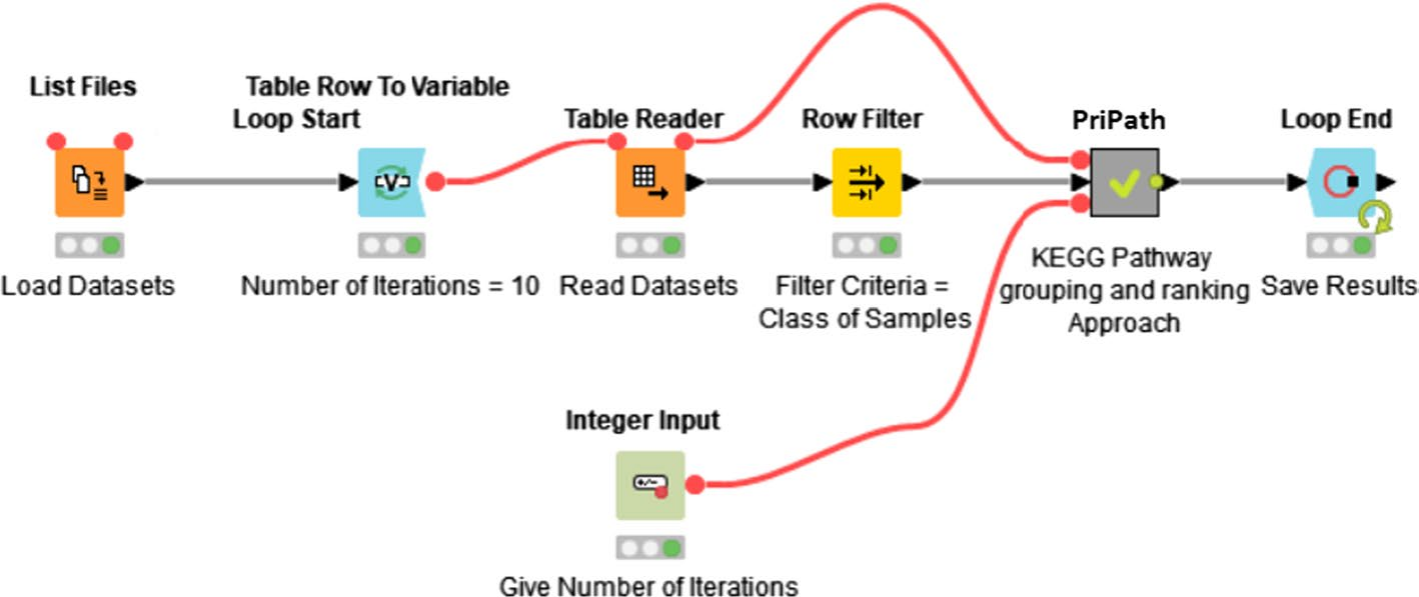
Overview of the PriPath KNIME workflow. The workflow contains programming structures such as loops (shown in blue), input nodes (shown as orange boxes), and workflow parameters (green box). Meta-nodes, shown in gray, encapsulate sub-workflows to increase modularity and readability. The green dots under the nodes demonstrate that the process has been successfully executed

The workflow of PriPath is shown in Fig. 2. The “List Files” node collects the data needed for PriPath analysis. The “Table Reader” node reads each file and forwards it to the PriPath algorithm, which is implemented as a sub-workflow in the PriPath meta-node. The node “Loop End” task is to collect all the results, which are then stored in Excel in the last node of the workflow (not shown). The “Integer Input” allows users to conveniently set various parameters, such as the number of cross-validations.

### Assessment of the PriPath predictions

#### Other tools that employ embedded feature selection

PriPath performs embedded feature selection. Therefore, it is similar to previous approaches we have developed, such as maTE and CogNet. CogNet relies on another tool called pathfindR that serves as the biological grouping tool to allow the main algorithm to rank active subnetworks of KEGG pathways. This grouping results in an enrichment analysis modeling networks, not just significant genes. Unlike CogNet, PriPath uses KEGG pathway knowledge without another grouping factor. The maTE tool was designed to utilize microRNAs and their target genes for grouping, representing a completely different type of information. In PriPath, we have included the knowledge of KEGG pathways in the grouping component. PriPath is different from SVM-RCE in the way that the genes are grouped. SVM-RCE clusters genes based on the k-means clustering algorithm. The outcome is a list of significant genes that survived the RCE procedure. This list has no prior biological knowledge compared to the other algorithms PriPath.

Comparing these tools is possible by assessing the performance values they report. To evaluate the differences among these four tools, which employ embedded feature selection, we have recorded the AUC values over the top 10 groups ranked by the scoring stage for each tool, except for SVM-RCE. For SVM-RCE, we measured the performance starting with 1000 genes and 100 clusters and decreased by 10% at each iteration. We used the final 10 clusters of SVM-RCE for comparison. This assessment is valid because all employed embedded feature selection and were run on the same datasets.

#### Other approaches to gene set enrichment

As discussed above, several other approaches aim to functionally enrich a set of differentially expressed genes [10]. We selected a few gene set enrichment tools widely used in literature. It is beyond the scope of this work to discuss all existing approaches, but for assessing the predictions, we have selected two popular enrichment tools. These tools are DAVID (version 6.8) and Reactome (version 78—Pathway Browser version 3.7. To assess these tools, 1000 genes were selected with a t-test. Then, the selected 1000 genes were submitted to DAVID and Reactome. The top 10 predicted KEGG pathways of these tools were recorded. Finally, we assessed the number of shared pathways among the predictions of these tools with the predictions by PriPath.

##### DAVID

Among many other functions of DAVID (https://david.ncifcrf.gov/tools.jsp), it offers gene set enrichment analysis. The tool accepts a set of genes as input but does not consider quantitative information such as gene expression. Another notable functionality of DAVID is its ability to translate among identifiers, for example, between Unigene IDs and GenBank IDs. We provided filtered gene sets to DAVID, equal to the combined training and testing data used during the model training of PriPath. DAVID then returned its interpretation of the datasets [39]. We applied a t-test to each gene expression data set in this study. We provided the top 1000 genes from the t-test results as inputs (ranked by increasing *p* value). Default values were used for DAVID analysis.

##### Reactome

Reactome (https://reactome.org/) provides bioinformatics tools for the interpretation, visualization, and analysis of pathway knowledge to assist in modeling, genome analysis, and systems biology. The constantly increasing size of the data samples is one of the main problems from a performance point of view [40]. This study applied a t-test to each gene expression data set. The top 1000 genes, according to the t-test, were used as input for Reactome. The default settings of Reactome were left unchanged. DAVID was used to translate between Reactome pathways and KEGG pathways.

## Results

### Performance evaluation of PriPath

**Fig. 3:**
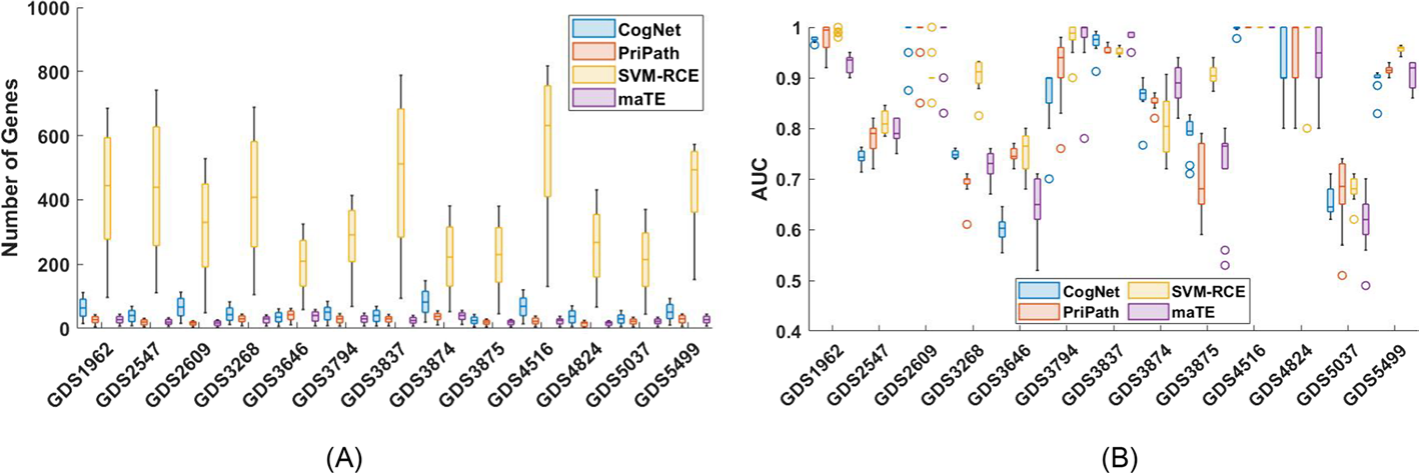
Performance evaluations of PriPath, CogNet, maTE, and SVM-RCE. **A** the number of genes, and **B** AUC values of 4 competing tools for the 13 selected datasets (top 10 results)

Gene expression and differential gene expression (DGE) analyses provide insights into the molecular mechanisms underlying the difference among phenotypes (e.g., control and disease). However, genes do not act alone, which makes it necessary to consider sets of genes to delineate among phenotypes. We introduce PriPath, which considers KEGG pathways as the grouping factor for expressed genes and thereby integrates the DGE analysis results of multiple genes. PriPath is freely available as a KNIME workflow. KEGG pathways and gene expression data must be available to the KNIME workflow, and examples are available in our GitHub repository. The output is a Microsoft Excel spreadsheet containing averaged performance metrics of ten cross-validations and a per-gene average (Additional file 2: Table S1). The PriPath workflow can be modified to provide information at all calculation steps, but we decided to provide only vital information. PriPath was used to evaluate 13 different gene expression data sets. The AUC results of this analysis, considering the number of groups (or clusters), are presented in Fig. 3A and Additional file 2: Table S1. The number of genes that were used is displayed in Fig. 3B. The average number of genes for ten iterations of MCCV is shown in column “#G” (Additional file 2: Table S1).

### Comparative evaluation with other tools that employ embedded feature selection

We previously observed that although CogNet, maTE, and SVM-RCE employ embedded feature selection, these tools perform differently (Fig. 3A). The mean AUC values of the four tools and their prediction for the 13 datasets are presented in Fig. 3A. In Fig. 3B, we plot the average number of genes associated with the prediction. As illustrated in Fig. 3A, PriPath performs similarly, but not the same, with maTE, CogNet, and SVM-RCE for different datasets. As shown in Fig. 3A, it uses far fewer genes than SVM-RCE and CogNet but a comparable number with maTE. Figure 3 implies that, on average, PriPath outperforms maTE by 1.2% while producing similar results with SVM-RCE. Regarding the average number of genes, SVM-RCE uses a 16-fold larger number than PriPath, with a lower number of genes leading to a more precise prediction. Note that the grouping factors in maTE and PriPath are different, which could be employed synergistically in the future.

### Comparative evaluation with functional enrichment tools

**Fig. 4:**
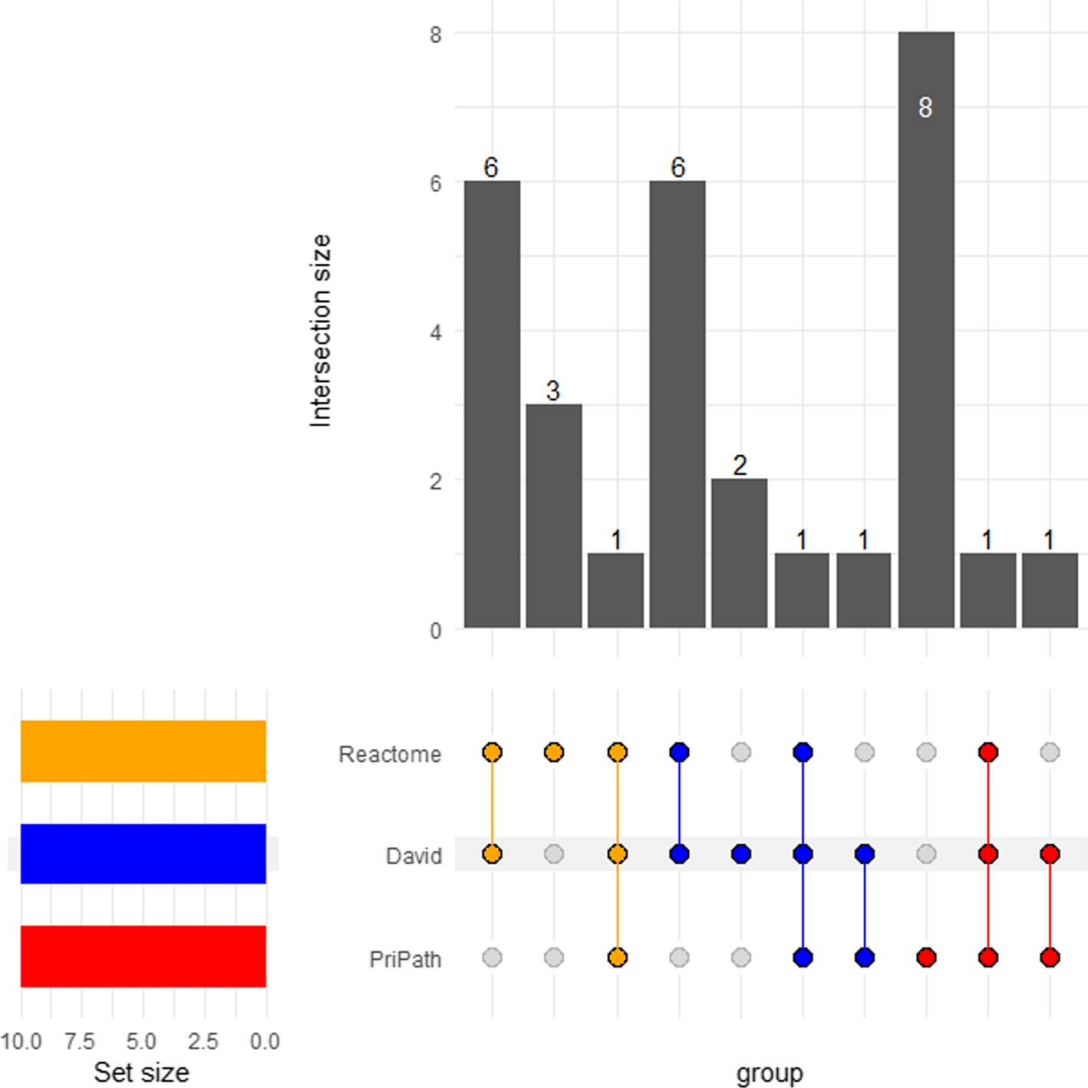
Comparative evaluation of PriPath against enrichment analysis results by Reactome and DAVID for the GDS1962 dataset. Results for the other datasets are available in Additional file 1: Figures S1–S13

Additionally, we compared the performance of PriPath with the enrichment tools, applying statistical analysis to gene expression datasets and identifying overrepresented pathways. Figure 4 displays the comparative evaluation of PriPath with these enrichment tools (i.e., DAVID and Reactome) for the GDS1962 dataset using a UpSetR plot [41]. An UpSetR plot comprises two axes and a connected-dot matrix. The vertical rectangles illustrate the number of elements in each list combination. The connected-dots matrix shows which combination of lists corresponds to which vertical rectangle. The horizontal bars that correspond to the size of sets indicate the participation of objects (from the vertical rectangles) in the respective lists [42].

**Fig. 5:**
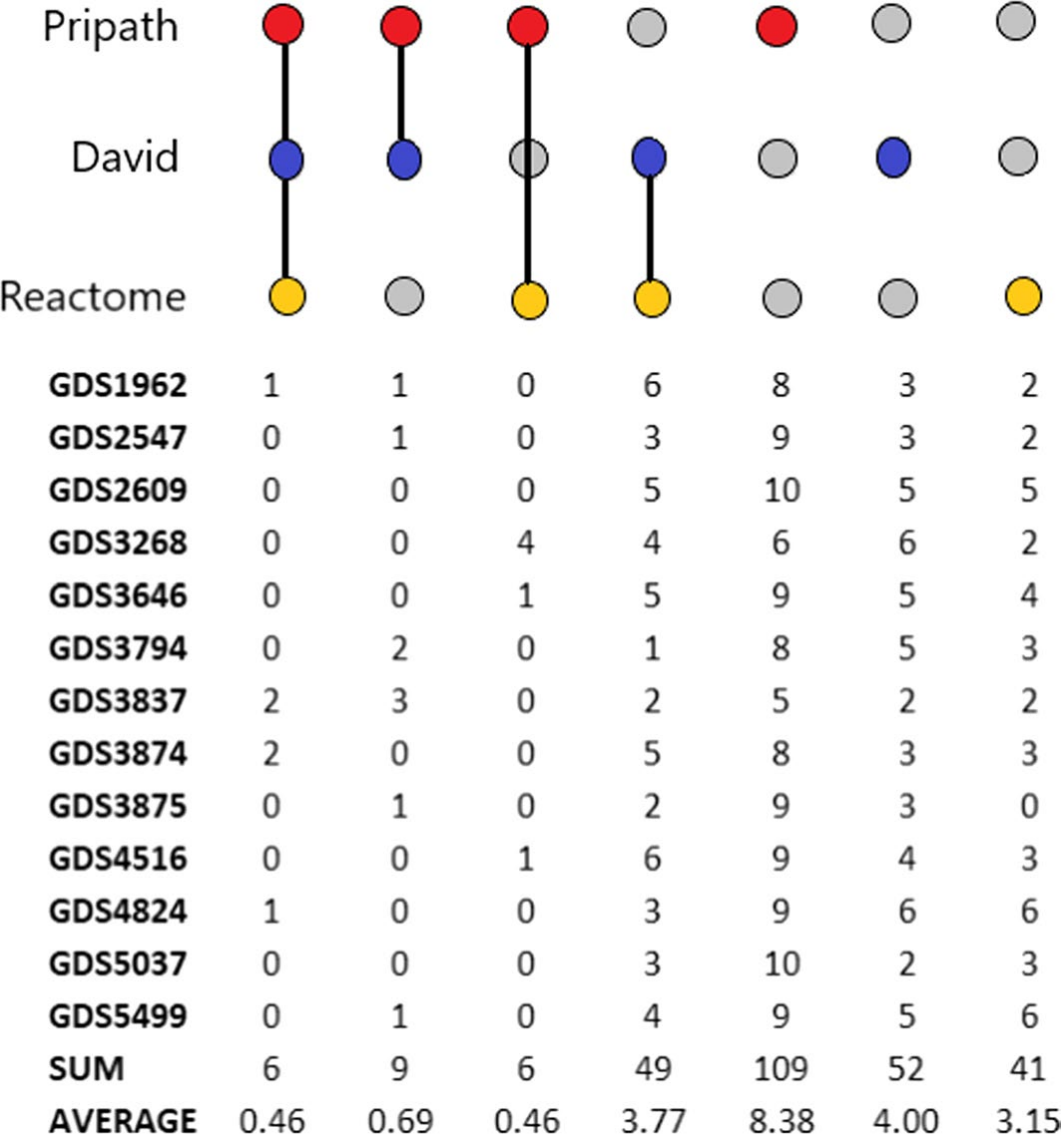
Sum and averages of the number of common KEGG pathways predicted among tools and the number of KEGG pathway predictions that are not shared for the 13 datasets

An example is illustrated in Fig. 4 for the GDS1962 dataset. In this example, Reactome and DAVID identify seven KEGG pathways in their prediction, and they identify one pathway in common with PriPath. PriPath shares another predicted pathway with Reactome and features eight unique pathways not predicted by the other two approaches. When the predictions are analyzed for 13 different datasets (Individual UpSetR plots can be found as Additional file 1: Figs. S1–S13), it can be observed that DAVID and Reactome predictions are more similar to each other than to PriPath’s. Figure 5 summarizes the individual results for all 13 datasets. PriPath shares 1–3 predictions with DAVID (average, 0.7); 1–4 predictions with Reactome (average, 0.5); and features unique pathways not predicted by the other two approaches (8.4 on average). Reactome and DAVID generally share 1–6 pathways (average, 3.8). PriPath shares 1–2 pathways with both tools (0.5 on average).

In some cases, PriPath does not share any predictions with either DAVID or Reactome. As pointed out above, the ground truth is unavailable for the datasets considered. A true comparison cannot be made without ground truth. Therefore, this benchmarking indicates that DAVID and Reactome work more similarly than PriPath. Hence PriPath provides alternative explanations to the data analysis. On the other hand, both Reactome and DAVID have predictions that are not shared with the other tool (4.7 on average for DAVID and 3.6 for Reactome). A more detailed assessment of the PriPath predictions is presented in the “Discussions” Section, which evaluates whether the predictions align well with the literature.

## Discussions

In the previous section, we presented the results of our experiments using PriPath on 13 different datasets: and our comparative evaluation with other tools employing embedded feature selection. In this section, we discuss the biological relevance of the PriPath predictions.

For each of the 13 selected datasets, we assess the top three predictions of PriPath. The assessment is done by manually checking whether any support for the prediction can be found in the literature. Support for the prediction means that the disease annotated for the dataset has been experimentally implicated with the prediction in the literature. We also assessed whether DAVID and Reactome predicted these top three pathways. Table 3 summarizes the association of the top three identified pathways of PriPath with the disease under study and presents whether these top three identified pathways were included in the top three predictions of DAVID and Reactome.

**Table 3:** Association of the top three predicted pathways of PriPath with the disease annotated for the study

Dataset accession	Annotated disease	KEGG Pathway ID	DAVID	Reactome	Pathway Name	Literature support for top 3 identified pathways by PriPath
GDS1962	Glioma	hsa05165	–	–	Human papillomavirus (HPV) infection	The presence of HCMV and HPV has been shown in gliomas. According to one study, HPV infection did not have a significant effect on the prognosis of glioma patients, while another study supports the presence of HPV in gliomas
		hsa04550	–	–	Signaling pathways regulating pluripotency of stem cells	None found
		hsa05131	–	–	Shigellosis	It can be an opportunistic infection in cancer patients undergoing chemotherapy
GDS2547	Prostate cancer	hsa04910	–	–	Insulin signaling pathway	Insulin resistance index is positively correlated with prostate volume in benign prostatic hyperplasia complicated with diabetes patients
		hsa03010	+	–	Ribosome	Ribosome-targeting drugs may be effective against diverse prostate cancer
		hsa05171	–	–	Coronavirus disease	Some research points to a possible liaison between prostate cancer (PCa) and COVID-19
GDS2609	Colorectal cancer	hsa04010	–	–	MAPK signaling pathway	Activation signaling pathways including the MAPK pathway enhance colorectal cancer progression
		hsa04657	–	–	IL-17 signaling pathway	IL-17A inhibitors have been assessed for their therapeutic and preventative potential in human cancers, particularly in colorectal cancer
		hsa05130	–	–	Pathogenic Escherichia coli infection	Pathogenic *E. coli* could be a factor in developing colorectal cancer
GDS3268	Colitis	hsa04151	–	+	PI3K-Akt signaling pathway	Up-regulation of the PI3K/Akt-mTOR signaling pathway can trigger cell apoptosis and inflammation in ulcerative colitis
		hsa05200	–	+	Pathways in cancer	It is known that long duration of ulcerative Colitis is a risk factor for the development of Colitis associated cancers
		hsa05164	–	–	Influenza A	Infection with influenza A could cause hemorrhagic colitis
GDS3646	Celiac disease	hsa05010	–	–	Alzheimer disease	Several types of dementia such as Alzheimer’s, vascular dementia, frontotemporal dementia have been association with Celiac disease
		hsa04020	–	–	Calcium signaling pathway	None found
		hsa05012	–	–	Parkinson disease	PARK7 plays an important role in the preservation of mucosal integrity in Celiac disease
GDS3794	Arthritis	hsa04620	–	–	Toll-like receptor signaling pathway	Rheumatoid arthritis (RA) development can be induced by the activation of the Toll-like receptor (TLR) signaling pathway
		hsa04657	–	–	IL-17 signaling pathway	The IL-17 cytokines play a crucial role in the chronic inflammation of the synovium in psoriatic arthritis
		hsa05022	–	–	Pathways of neurodegeneration—multiple diseases	Neurodegenerative disease increases the progress of arthritis
GDS3837	Lung cancer	hsa04974	+	–	Protein digestion and absorption	None found
		hsa04510	–	–	Focal adhesion	FAK is significant in small cell lung cancer biology and targeting its kinase domain may have therapeutic potential
		hsa04151	+	+	PI3K-Akt signaling pathway	FGF21 may function as a tumor promotor by activating the SIRT1/PI3K/AKT signaling pathway in lung cancer
GDS3874	Diabetes	hsa05203	–	–	Viral carcinogenesis	None found
		hsa04625	–	–	C-type lectin receptor signaling pathway	None found
		hsa05166	–	–	Human T-cell leukemia virus 1 infection	None found
GDS3875	Diabetes	hsa05168	–	–	Herpes simplex virus 1(HSV-1) infection	HSV-1 infection has an important association with type 2 diabetes
		hsa04910	–	–	Insulin signaling pathway	Insulin resistance, is generated by the disruption of the insulin signaling pathway
		hsa05022	–	–	Pathways of neurodegeneration—multiple diseases	One of the conditions which result in neurodegeneration is diabetes
GDS4516 GDS4718	Colorectal cancer (CRC)	hsa04080	–	–	Neuroactive ligand-receptor interaction	None found
		hsa04721	–	–	Synaptic vesicle cycle	None found
		hsa04724	–	–	Glutamatergic synapse	Neuroligin1 is the main component of excitatory glutamatergic synapses complex is verified as a new poor prognostic marker for CRC
GDS4824	Prostate cancer	hsa04080	–	–	Neuroactive ligand-receptor interaction	None found
		hsa05163	–	–	Human cytomegalovirus infection	The activation of the Human cytomegalovirus (HCMV) major immediate early promoter by androgen in the prostate might contribute to oncomodulation in prostate cancers
		hsa04062	–	–	Chemokine signaling pathway	Chemokines play modulatory roles in prostate cancer metastasis
GDS5037	Asthma	hsa04530	–	–	Tight junction (TJ)	Asthma may be linked to differential expression of TJ
		hsa05016	–	–	Huntington’s disease	None found. However, similarly to hsa05022 below, it leads to neurodegeneration
		hsa05022	–	–	Pathways of neurodegeneration—multiple diseases	Asthma, especially when severe, is associated with features of neuroinflammation and neurodegeneration
GDS5499	Pulmonary Hypertension	hsa04010	–	–	MAPK signaling pathway	Inhibition of the MAPK axis could prevent vascular remodeling in pulmonary artery hypertension
		hsa04621	–	–	NOD-like receptor signaling pathway	NOD-like receptor subfamily C3 may potentially be a marker for PH patients
		hsa04390	–	–	Hippo signaling pathway	Pulmonary arterial hypertension is ameliorated by suppressing the HIPPO signaling pathway

The table shows the accession of the dataset and the disease label

DAVID and Reactome columns show whether the PriPath prediction was also in the top three predictions of these tools. The pathway name is provided so the literature assessment of the top three PriPath predictions can be better appreciated. The references to the literature support are provided within the text following the table

In the following, we provide more details for the literature assessment of the predictions presented in Table 3.

For the GDS1962 dataset, a study concerning glioma, PriPath’s top predictions are Human papillomavirus infection (hsa05165), Signaling pathways regulating pluripotency of stem cells (hsa04550), and Shigellosis (hsa05131). The association between viruses and cancer has often been shown, and the correlation between HPV and glioma is not an exception [43]. Shigellosis is also an opportunistic infection of the immune-compromised, so it could be seen in cancer patients undergoing chemotherapy. The other tested tools did not predict these pathways within the top three. With this statement, we do not want to dismiss the predictions by DAVID and Reactome. Both tools predict Calcium signaling among their top predictions, which has a link to glioma [44], and proteoglycans in cancer, for which we could not find a basis in the literature. DAVID predicts neuroactive ligand-receptor interaction, which has been implicated in glioma [45], and Reactome suggests retrograde endocannabinoid signaling, which has also been linked to glioma [46]. Hence, it seems vital to perform predictions with multiple tools to comprehensively understand any dataset. In the following, we will only analyze the literature support for the PriPath predictions. However, we assume that DAVID and Reactome predict KEGG pathways with similar literature support as PriPath.

For the GDS2547 dataset, a study concerning prostate cancer, PriPath’s top predictions are insulin signaling pathway (hsa04910), Ribosome (hsa03010), and Coronavirus disease (hsa05171). The connection between diabetes and prostate cancer has been demonstrated in [47]. In the literature, it has been shown that Ribosome-targeting drugs may be effective against diverse prostate cancer [48]. Some research findings point to a possible hidden liaison between prostate cancer (PCa) and COVID-19 [49]. DAVID also predicted associations between the data and the Ribosome pathway in its top 3 predictions.

For the GDS2609 dataset, a study concerning colorectal cancer, PriPath’s top predictions are MAPK signaling pathway (hsa04010), IL-17 signaling pathway (hsa04657), and pathogenic *Escherichia coli* infection (hsa05130). The relationship between the MAPK signaling pathway and Colorectal cancer has been illustrated in [50]. Research shows that IL-17A inhibitors have preventive potential in human cancers, particularly colorectal cancer [51]. Pathogenic *E. coli* could also contribute to developing colorectal cancer [52]. The other tested tools did not predict these associations between the data and the investigated disease in their top three predictions.

For the GDS3268 dataset, a study concerning Colitis, PriPath’s top predictions are the PI3K-Akt signaling pathway (hsa04151), pathways in cancer (hsa05200), and Influenza A (hsa05164). The association between the PI3K-Akt signaling pathway and Colitis has been shown in [53]. The long duration of Ulcerative Colitis is known as a risk factor for the development of Colitis associated cancers [54]. The same study reported that the analysis of the transcriptomic changes in the colonic mucosa of long-duration Ulcerative Colitis patients revealed colitis-associated cancer pathways. Infection with influenza A could cause hemorrhagic colitis [55]. Reactome predicted the association between the data and PI3K-Akt signaling pathway and pathways in cancer in its top three predictions.

For the GDS3646 dataset, a study concerning Celiac disease (CD), PriPath’s top predictions are Alzheimer’s disease (hsa05010), Calcium signaling pathway (hsa04020), and Parkinson’s disease (hsa05012). Several types of dementia, such as Alzheimer’s, vascular dementia, and frontotemporal dementia, were reported in association with CD [56]. Lurie et al. also reported Alzheimer’s disease in two patients diagnosed with CD after 60 years [57]. The association between Parkinson’s disease and Celiac has been shown in [58]. The other tools did not report these pathways in their top three predictions for this dataset.

For the GDS3794 dataset, a study concerning Arthritis, PriPath’s top predictions are Toll-like receptor signaling pathway (hsa04620), IL-17 signaling pathway (hsa04657), and pathways of neurodegeneration—multiple diseases (hsa05022). Rheumatoid arthritis (RA) is associated with the Toll-like receptor (TLR) signaling pathway [59]. The IL-17 cytokines play an important role in the chronic inflammation of the synovium in Psoriatic arthritis [60]. Research showed that neurodegenerative disease increases the progression of arthritis [61]. The other tested tools did not contain these pathways within their top three predictions.

For the GDS3837 dataset, a study concerning lung cancer, PriPath’s top predictions are protein digestion and absorption (hsa04974), focal adhesion (hsa04510), and PI3K-Akt signaling pathway (hsa04151). The association between Focal adhesion and lung cancer has been shown [62]. The dysregulation of the PI3K-Akt signaling pathway is known to affect lung cancer [63]. DAVID and Reactome predicted the associations between the data and the PI3K-Akt signaling pathway in their top three predictions. DAVID further suggested the protein digestion and absorption pathway in its top three.

For the GDS3874 dataset, a study concerning Diabetes, PriPath’s top predictions are viral carcinogenesis (hsa05203), C-type lectin receptor signaling pathway (hsa04625), and Human T-cell leukemia virus 1 infection (hsa05166). There is no study about the association between Diabetes and our predicted pathways for this dataset. The dataset GDS3874 shows a slight variance in PriPath performance (Fig. 3A). Perhaps Diabetes shares some commonly dysregulated genes with the predicted pathways which have not been reported. The other tested tools did not identify these pathways in their top 3 predictions for this dataset.

For the GDS3875 dataset, which is another study on Diabetes, PriPath’s top predictions are Herpes simplex virus 1 infection (hsa05168), Insulin signaling pathway (hsa04910), and pathways of neurodegeneration—multiple diseases (hsa05022). HSV-1 infection has an important association with diabetes, as explained in [64]. This disease, also known as insulin resistance, is generated by disrupting the insulin signaling pathway [65]. Additionally, one of the conditions which result in neurodegeneration is diabetes [66]. The other tested tools did not detect these pathways in their top three predictions.

For the GDS4516 dataset, a study concerning colorectal cancer (CRC), PriPath’s top predictions are neuroactive ligand-receptor interaction (hsa04080), synaptic vesicle cycle (hsa04721), and glutamatergic synapse (hsa04724). The association between the glutamatergic synapse pathway and colorectal cancer has been reported, and glutamatergic synapse has recently been verified as a new prognostic marker for CRC [67]. The other tested tools didn’t return these pathways in their top three predictions.

For the GDS4824 dataset, a study concerning prostate cancer, PriPath’s top predictions are neuroactive ligand-receptor interaction (hsa04080), Human cytomegalovirus infection (hsa05163), and chemokine signaling pathway (hsa04062). Proteins in the neuroactive ligand-receptor interaction pathway are hubs in the protein–protein interaction network and play roles in prostate cancer [68]. The association between prostate cancer and Human cytomegalovirus infection has been demonstrated [69]. Chemokines play modulatory roles in prostate cancer metastasis [60, 70]. The other tools did not identify these pathways in their top three predictions.

For the GDS5037 dataset, a study concerning Asthma, PriPath’s top predictions are tight junction (TJ; hsa04530), Huntington’s disease (hsa05016), and pathways of neurodegeneration—multiple conditions (hsa05022). Asthma may be linked to the differential expression of TJ, as reported in [71]. Asthma, especially when severe, is associated with features of neuroinflammation and neurodegeneration [72]. The other tested tools did not report these pathways in their top 3 predictions.

For the GDS5499 dataset, a study concerning pulmonary hypertension, PriPath’s top predictions are MAPK signaling pathway (hsa04010), NOD-like receptor signaling pathway (hsa04621), and Hippo signaling pathway (hsa04390). Research has shown that inhibiting the MAPK axis could prevent vascular remodeling in pulmonary artery hypertension [73]. A NOD-like receptor signaling pathway has been previously associated with pulmonary hypertension [74]. PAH has been observed to suppress the HIPPO signaling pathway [75]. The other tested tools did not return these pathways in their top 3 predictions.

In summary, for most datasets (except GDS3874), the top 3 pathways predicted by PriPath have been associated with the diseases of the respective dataset in the literature. Hence, we have shown that PriPath successfully identified dysregulated pathways of various illnesses that seem to have a basis in molecular biology.

PriPath, CogNet, maTE, and SVM-RCE tools integrate biological information into the machine learning algorithm and help scientists to understand disease mechanisms at the molecular level and to generate hypotheses. These tools natively analyze different aspects (e.g., maTE analyzes the microRNA-target portion of the dysregulated genes). As presented in “Comparative Evaluation with other tools that employ embedded feature selection” Section, PriPath was compared with these tools, which implement different data integration approaches but are also based on the G–S–M model. Hence, it becomes possible to compare the performance metrics that these tools report. All methods were tested with 13 different gene expression data sets. Our experiments showed that PriPath utilizes fewer genes than SVM-RCE. On average, PriPath outperformed maTE by 1.2% while producing similar results with SVM-RCE. SVM-RCE used a 16-fold larger number of genes than PriPath in terms of the mean number of genes, making the PriPath predictions favorable.

Currently, the standard approach to differential gene expression analysis is to provide a list of genes expressed significantly different among conditions. The main aim of PriPath is to discover KEGG pathways that have a biological role in the disease under investigation. Such associations are found when several differentially expressed genes support a KEGG pathway. Other approaches, such as statistical ones, are presented by Reactome and DAVID. A fair comparison with these methods is impossible since they use different approaches and because there needs to be ground truth data to compare the outcomes. We did check whether DAVID and Reactome provide the same KEGG pathways as PriPath. Our analysis shows a larger overlap between DAVID and Reactome but little overlap with PriPath regarding predictions (Fig. 5). This indicates that PriPath functions differently than the other tools. In the absence of ground truth for this type of data, it is impossible to discard the prediction of any of these tools. Therefore, we assessed whether the PriPath predictions make sense on the biological level. For many datasets, the pathways predicted by PriPath have support in the literature for the annotated disease of the dataset. Thus, we have observed that PriPath successfully identified dysregulated pathways of various illnesses that seem to have a basis in molecular biology. This should not discard Reactome and DAVID predictions, and a researcher that may want to discover significantly dysregulated pathways could integrate several approaches of gene set enrichment.

## Conclusions

Dysregulation of gene expression is a hallmark of diseases. Determining dysregulation on the transcriptional level can be performed using high throughput arrays, sequencing approaches, etc. Such analyses result in a set of genes that are differentially expressed among different conditions. Correlating this information with prior biological knowledge can show how differential gene expression drives a disease. We introduce a novel tool named PriPath that groups the biological information in KEGG pathways to analyze differential gene expression. PriPath uses a list of KEGG pathways and gene expression data to perform embedded feature selection that we refer to as the G–S–M method. PriPath produces performance metrics such as accuracy, sensitivity, specificity, and AUC describing the performance of the generated models. Thus, the best model can be selected. Additionally, PriPath presents a ranked list of important KEGG pathways that can explain the differential gene expression among the analyzed samples.

In summary, PriPath is a novel tool that complements predictions of other gene set enrichment tools and presents alternative explanations for observed differential gene expression. It is advisable to integrate the results of multiple such tools in practice to perform unbiased gene set enrichment.

## Supplementary Information

**Additional file 1.** Comparative Evaluation of PriPath with enrichment analysis provided by Reactome and DAVID for different datasets.

**Additional file 2. Supplementary Table 1.** PriPath performance metrics for 13 different datasets. AUC values for different numbers of clusters for each dataset; and the average number of genes used for each level for each dataset are shown.

## Data Availability

The PriPath Knime workflow is freely available: (1) https://github.com/malikyousef/PriPath.git; (2) https://kni.me/s/xdHXGrOP-D2eIvvi. All the datasets used in this study are publicly available at the gene omnibus at NCBI and can be retrieved using the cited accession numbers. Accession numbers of datasets (GDS1962, GDS2547, GDS4824, GDS3268, GDS3646, GDS3874, GDS3875, GDS5037, GDS5499, GDS3837, GDS4516, GDS4718, GDS2609, and GDS3794) are shown in the first column of Table 1.

## Abbreviations

ACC: Accuracy
AUC: Area under curve
COVID-19: Coronavirus disease
CRC: Colorectal cancer
DAVID: Database for annotation, visualization, and integrated discovery
FAIR: Findable, accessible, interoperable, and reusable
FAK: Focal adhesion kinase
FGF21: Fibroblast growth factor 21
FN: False negative
FP: False positive
GEO: Gene expression omnibus
GO: Gene ontology
G–S–M: Grouping, scoring, and modeling
HCMV: Human cytomegalovirus
HPV: Human papillomavirus
HSV-1: Herpes simplex virus 1
IL-17: Interleukin-17
KEGG: Kyoto encyclopedia of genes and genomes
KNIME: Konstanz information miner
MAPK: Mitogen-activated protein kinase
MCCV: Monte Carlo cross validation
miRNA: MicroRNA
ML: Machine learning
mRNA: Messenger-RNA
mTOR: Mammalian target of rapamycin
NCBI: National Center for Biotechnology Information
NOD: Nucleotide-oligomerization domain
non-TB: Non-topology-based
PAH: Pulmonary arterial hypertension
PARK7: Parkinsonism associated deglycase
PCa: Prostate cancer
PH: Pulmonary hypertension
PI3K-Akt: Phosphatidylinositol 3-kinase
RA: Rheumatoid arthritis
RF: Random forest
ROC: Receiver operator characteristic
SE: Sensitivity
SIRT1: Sirtuin 1
SP: Specificity
SVM-RCE: Support vector machines-recursive cluster elimination
SVM-RCE-R: Recursive cluster elimination-based rank function
SVM-RNE: Support vector machines with recursive network elimination
TB: Topology-based
TJ: Tight junction
TLR: Toll-like receptor
TN: True negative
TP: True positive
