# Supplementary material for: PriPath: identifying dysregulated pathways from differential gene expression via grouping, scoring, and modeling with an embedded feature selection approach
Source: BMC Bioinformatics. 2023 Feb 23;24:60. doi: 10.1186/s12859-023-05187-2 (PMC9947447; doi:10.1186/s12859-023-05187-2)
Supplement: Supplementary file 1 — Additional file 1. Comparative Evaluation of PriPath with enrichment analysis provided by Reactome and DAVID for different datasets. [file 12859_2023_5187_MOESM1_ESM.docx]

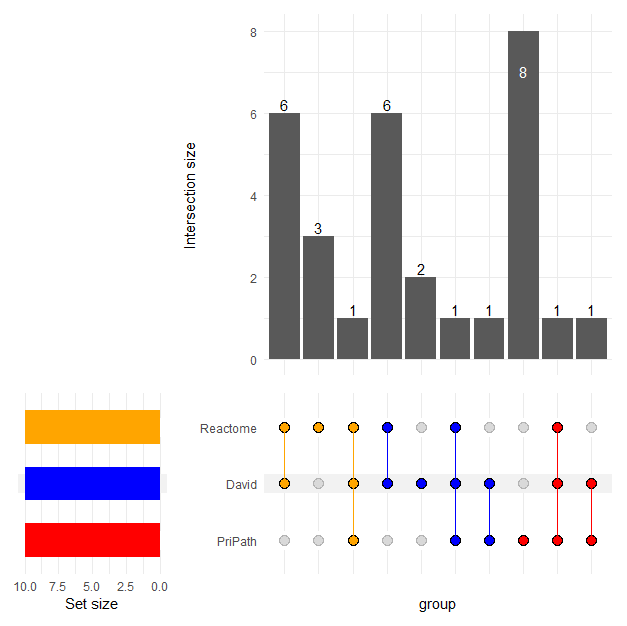


***Additional File 1.*** *Comparative Evaluation of PriPath with enrichment analysis provided by Reactome and DAVID for the GDS1962 dataset.*

*
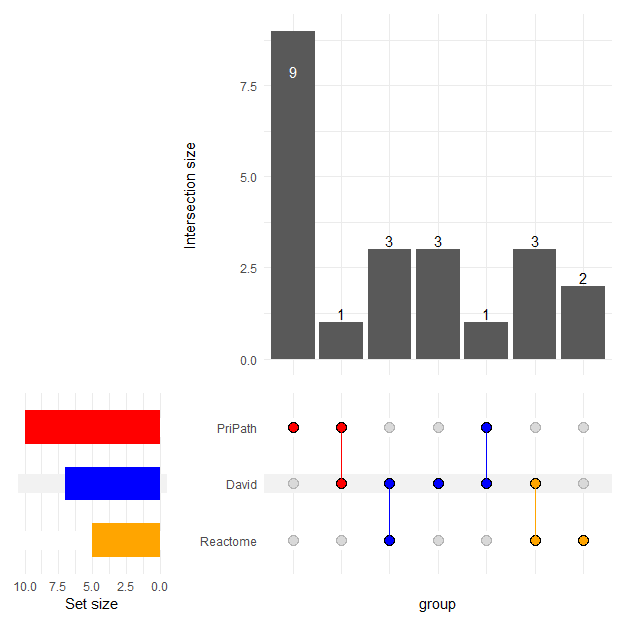
*

***Supplementary Figure 2.*** *Comparative Evaluation with traditional enrichment tools for GDS2547 dataset.*

*
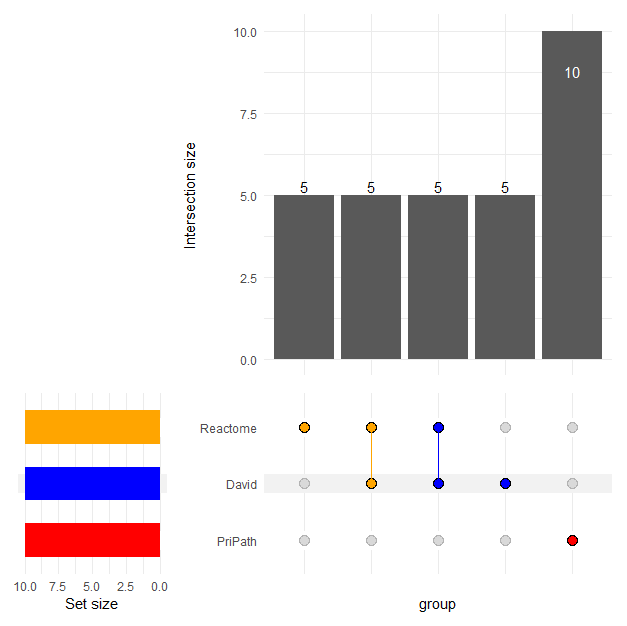
*

***Supplementary Figure 3.*** *Comparative Evaluation with traditional enrichment tools for GDS2609 dataset.*

*
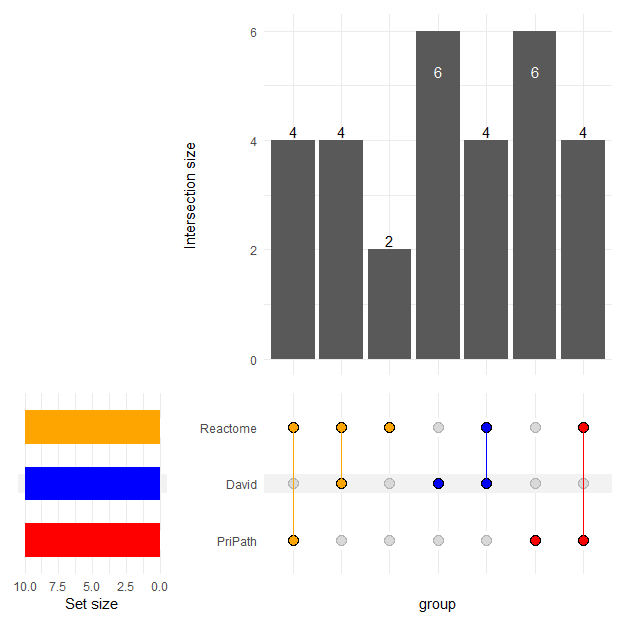
*

***Supplementary Figure 4.*** *Comparative Evaluation with traditional enrichment tools for GDS3268 dataset.*

*
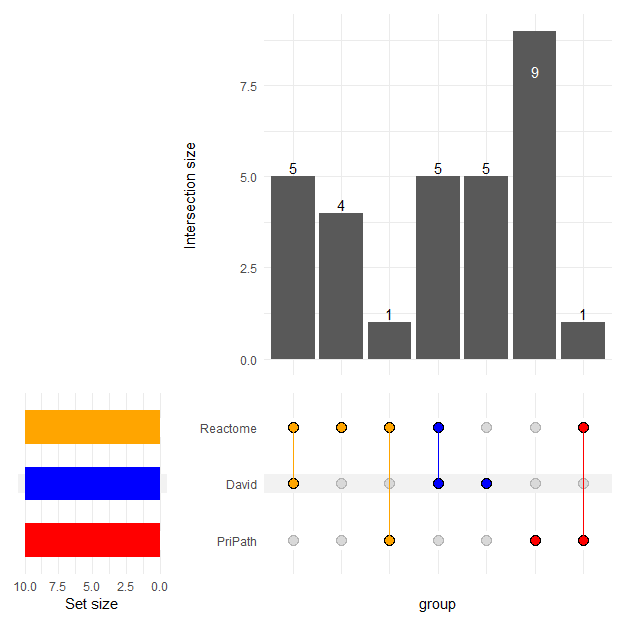
*

***Supplementary Figure 5.*** *Comparative Evaluation with traditional enrichment tools for GDS3646 dataset.*

*
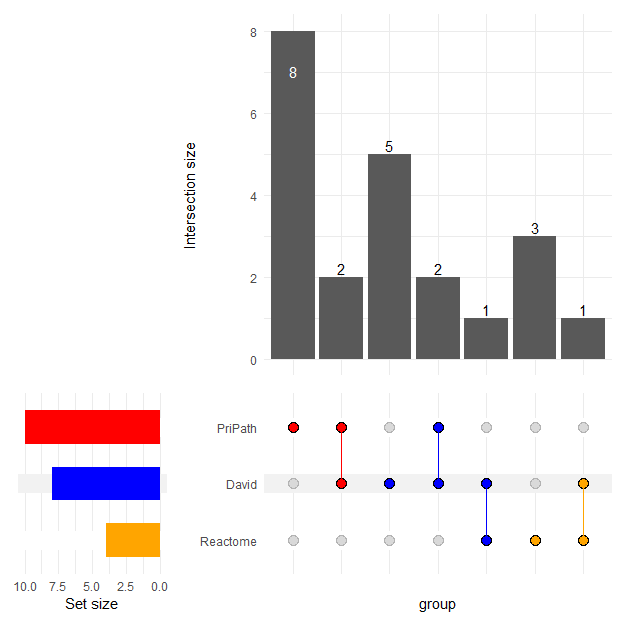
*

***Supplementary Figure 6.*** *Comparative Evaluation with traditional enrichment tools for GDS3794 dataset.*

*
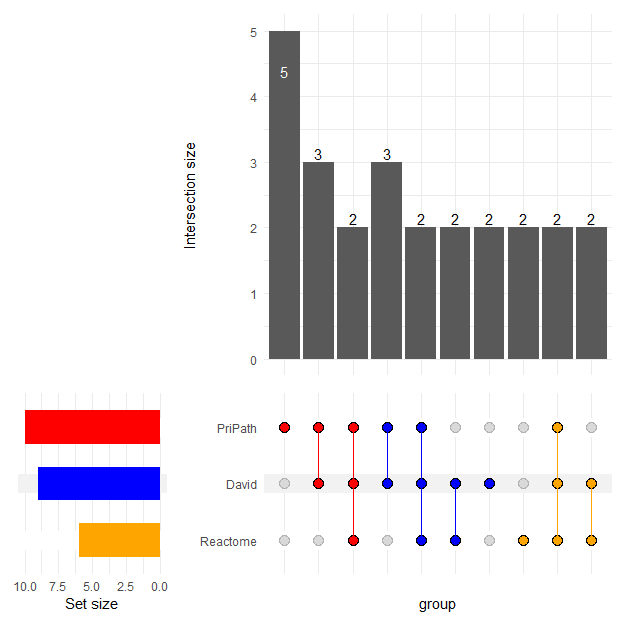
*

***Supplementary Figure 7.*** *Comparative Evaluation with traditional enrichment tools for GDS3837 dataset.*

*
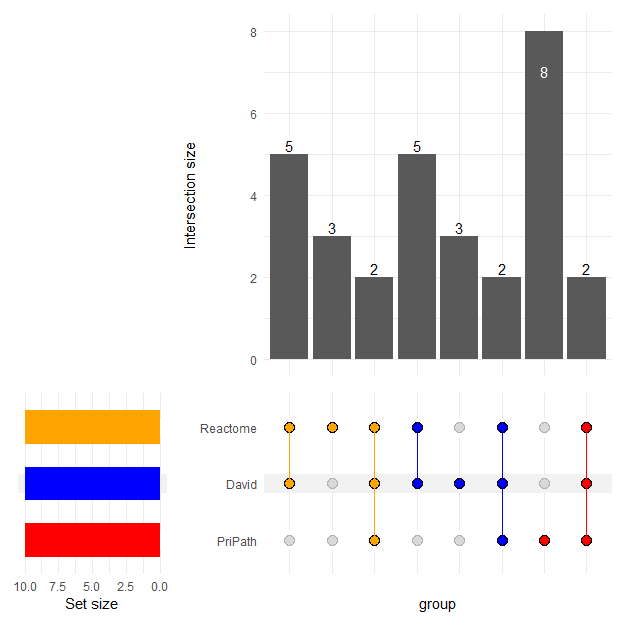
****Supplementary Figure 8.*** *Comparative Evaluation with traditional enrichment tools for GDS3874 dataset.*

*
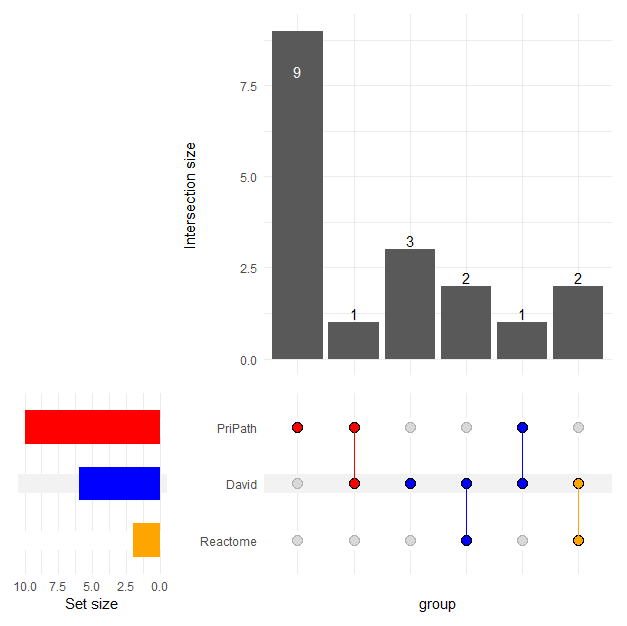
*

***Supplementary Figure 9.*** *Comparative Evaluation with traditional enrichment tools for GDS3875 dataset.*

*
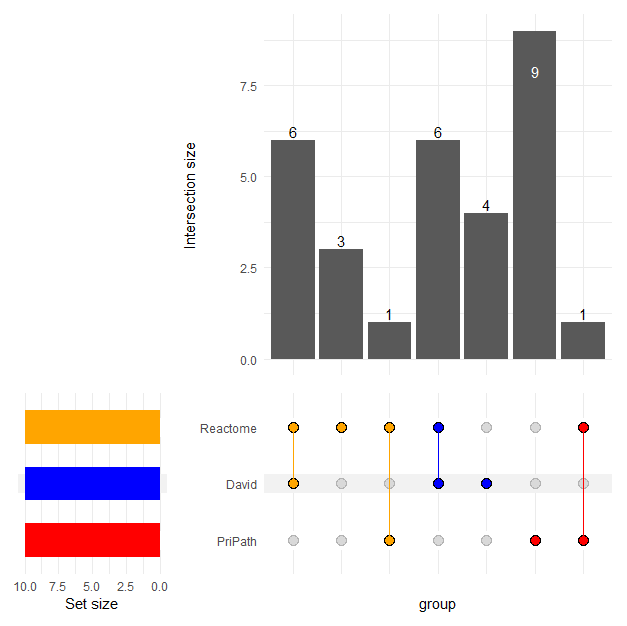
*

***Supplementary Figure 10.*** *Comparative Evaluation with traditional enrichment tools for GDS4516 dataset.*

*
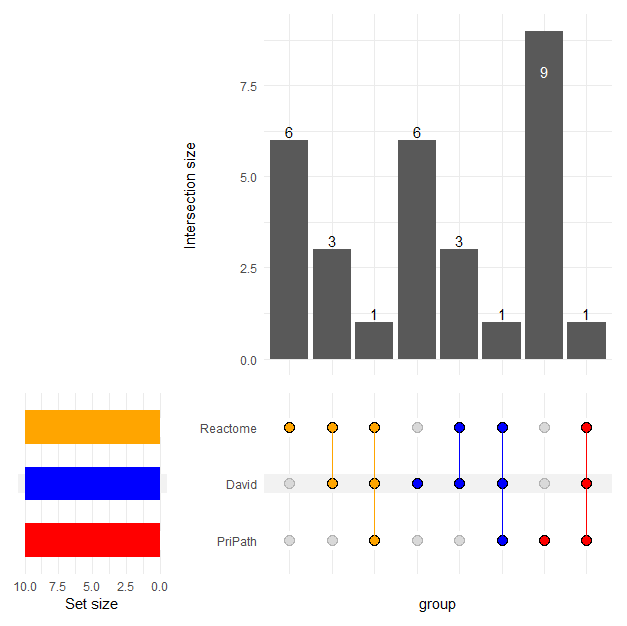
*

***Supplementary Figure 11.*** *Comparative Evaluation with traditional enrichment tools for GDS4824 dataset.*

*
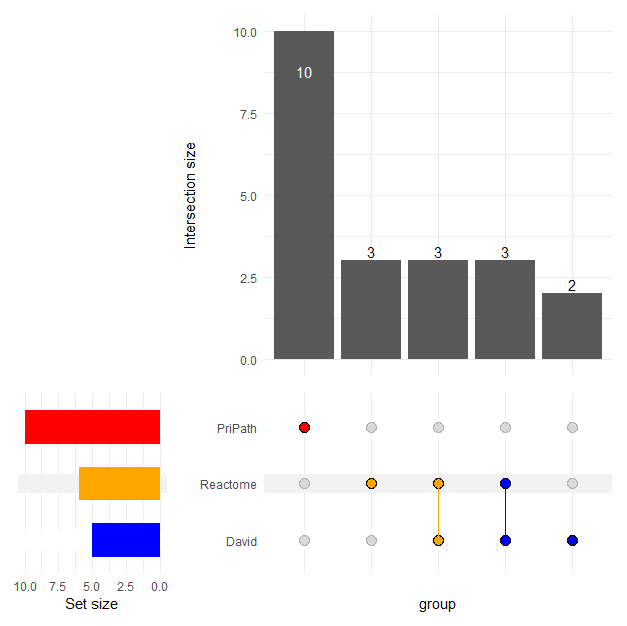
*

***Supplementary Figure 12.*** *Comparative Evaluation with traditional enrichment tools for GDS5037 dataset.*

*
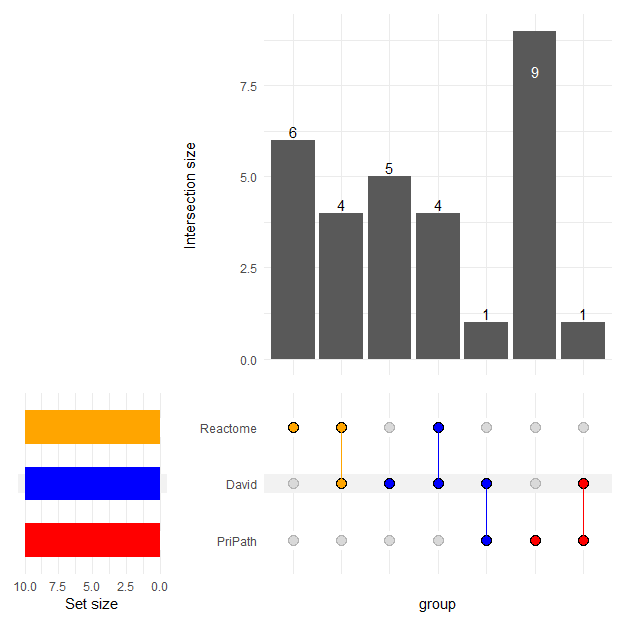
*

***Supplementary Figure 13.*** *Comparative Evaluation with traditional enrichment tools for GDS5499 dataset.*
