# Supplementary material for: PriPath: identifying dysregulated pathways from differential gene expression via grouping, scoring, and modeling with an embedded feature selection approach
Source: BMC Bioinformatics. 2023 Feb 23;24:60. doi: 10.1186/s12859-023-05187-2 (PMC9947447; doi:10.1186/s12859-023-05187-2)

***Supplementary Table 1.*** *PriPath performance metrics for 13 different datasets. AUC values for different numbers of clusters for each dataset; and the average number of genes used for each level for each dataset are shown.*


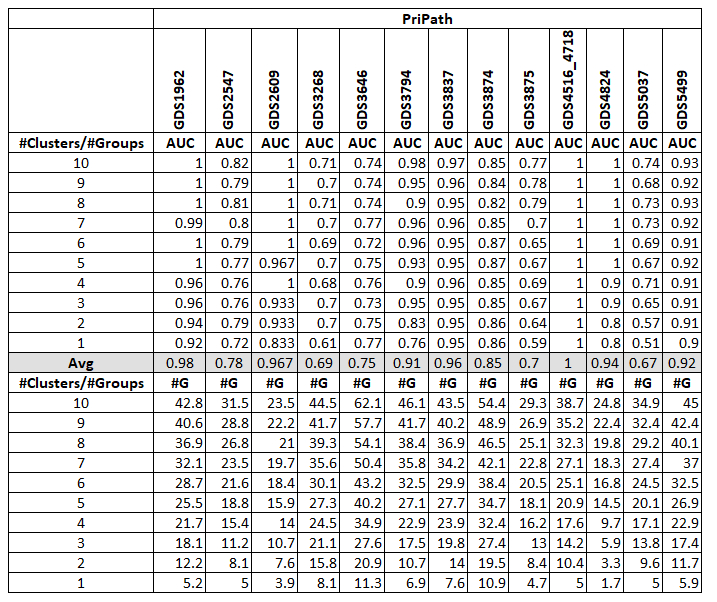

Supplement: Supplementary file 2 — Additional file 2. Supplementary Table 1. PriPath performance metrics for 13 different datasets. AUC values for different numbers of clusters for each dataset; and the average number of genes used for each level for each dataset are shown. [file 12859_2023_5187_MOESM2_ESM.docx]
